# Heavy Smoking Patients Receiving a Lung Cancer Screen Want to Quit: A Call for Tailored Cessation Interventions

**DOI:** 10.3389/fmed.2022.816694

**Published:** 2022-05-11

**Authors:** Michael H. Bernstein, Grayson L. Baird, Karim Oueidat, Saurabh Agarwal, Alexander Atalay, Shannon Healey, Terrance T. Healey

**Affiliations:** ^1^Department of Diagnostic Imaging, The Warren Alpert Medical School of Brown University, Providence, RI, United States; ^2^Department of Behavioral and Social Sciences, School of Public Health, Center for Alcohol and Addiction Studies, Brown University, Providence, RI, United States; ^3^Brown Radiology Human Factors Lab, Providence, RI, United States; ^4^Lifespan Biostatistics Core, Providence, RI, United States; ^5^Department of Radiology and Medical Imaging, University of Virginia Hospital Medical Center, Charlottesville, VA, United States; ^6^Rhode Island Medical Imaging, Providence, RI, United States

**Keywords:** smoking, lung cancer, lung cancer screening chest CT, substance use, smoking cessation, smoking attitudes

## Abstract

**Background:**

Lung cancer screening for current or former heavy smokers is now recommended among all asymptomatic adults 50–80 years old with a 20 pack-year history of smoking. However, little is known about the smoking-related attitudes of this population.

**Method:**

An assessment was conducted among 1,472 current smokers who presented for an annual lung cancer screen at one of 12 diagnostic imaging sites in Rhode Island between April 2019 and May 2020. Patients were asked about their use of smoking products, interest in quitting, and smoking-related attitudes.

**Results:**

Patients smoked a median of 16 cigarettes per day; 86.6% were daily cigarette smokers and 30.1% were daily cigar smokers. In total, 91.4% of patients were, to some degree, interested in quitting smoking and 71.4% were seriously thinking about quitting in the next 6 months or sooner. Patients planned on smoking less regardless of whether their lung screen was positive or negative for cancer, though they were more likely to plan on smoking less if negative (on 0–3 pt Likert scale: 0.31, 95% CI [0.27, 0.34] vs. 0.77, 95% CI [0.72, 0.81]). Confidence in quitting and belief in one’s inherent ability to quit smoking varied substantially within the sample.

**Conclusion:**

Nearly all current smokers receiving a lung cancer screen have some interest in smoking cessation. Due to the heterogeneity in some smoking-related attitudes, tailored interventions for this population should be tested.

## Introduction

Lung cancer is the second most prevalent type of cancer, and is responsible for 22.4% of all cancer deaths (more than any other cancer type) (1). The risk of developing lung cancer increases drastically with age and smoking status (2). Due in part to the benefit associated with providing annual screens, the US Prevention Services Task Force (USPSTF) recently expanded their recommendations low-dose computed tomography (LDCT) screening to include asymptomatic adults 50–80 years old with a 20 pack-year smoking history (3).

As more patients with a lengthy history of smoking receive lung cancer screens, and primary care providers increasingly view LDCTs as beneficial (4), greater attention should be given to screening as an opportunity to connect these patients with smoking cessation resources (5–7). According to one study, only a minority of participants who received a lung cancer screen received any smoking cessation resource (8). However, randomized trials of smoking cessation interventions administered during the course of lung screening have generally not been efficacious.

One possibility for the lack of efficacious cessation interventions is that interventions are not adequately tailored to this particular population of smokers. Those seeking lung cancer screens likely have a different psychological profile than smokers as a whole, since they are: older, heavy smokers, and health-conscious enough to undergo a cancer screen in the first place. To maximize the efficacy of cessation interventions in this population, a thorough understanding of their attitudes toward smoking cessation engagement is needed. There has been little research in this area. Existing studies have been qualitative and yielded somewhat discrepant findings (5, 9). In one study, Park et al. (15) interviewed 35 patients who received a lung cancer screen. Most of the participants did not indicate that health risk was a motivator for screening, and the authors conclude that “smoking cessation and prevention interventions during lung screening should explore risk perceptions, emotions, and quit confidence.” (p. 166). Large-scale quantitative studies are needed to examine smoking cessation interest in this population. Relatedly, more work is needed to examine other psychological factors among these patients such as self-efficacy to quit, perceived control over smoking behavior, and smoking cessation contemplation. Consistent with the recommendation from Park and colleagues, our objective is to address these gaps in the present study.

## Materials and Methods

### Procedure and Participants

This study was reviewed by the Lifespan Hospital system IRB and deemed not to need approval. An assessment of smoking behaviors and attitudes was conducted among 1,472 current smokers who presented for an annual lung cancer screen at one of 12 diagnostic imaging sites in Rhode Island between April 2019 and May 2020. Patients were eligible for a lung cancer screen if they were at least 30 pack-year smokers and 55–80 years old, consistent with guidelines at the time. Patients were given the assessment during intake. In total, 94% were White, 2.5% Black/African American, 0.8% American Indian or Alaskan Native, 1% Asian, and 2% Other; 5% identified as Hispanic.

### Measures

Smoking behavior was assessed by assessing the frequency participants smoked cigarettes, cigars, and e-cigarettes (every day, some days, not at all). Cigarettes smoked per day and the number of years smoked were also measured.

Interest in quitting was assessed with: “In general, how interested are you in quitting?” (from “Not at all interested” to “very interested”).

Planning to quit was assessed with “Are you seriously thinking of quitting smoking?” with response options of “Yes, within the next 30 days,” “Yes, within the next 6 months,” and “No, not thinking of quitting” (10).

Attitudes About Quitting was assessed with: (1) “How confident are you that you will not use tobacco in 1 year?” (from “Not very confident” to “very confident”); (2) “Do you believe you have the ability to change your smoking habits?” [from “not at all (I am totally unable to change my smoking habits)]” “to “Completely (I am totally able to change my smoking habits)]”; (3) The contemplation ladder (11).

Smoking behavior as a function of lung screen results was assessed by asking how smoking behavior would change if the lung screen indicates they do and do not have lung cancer (from “substantially less” to “substantially more”).

Attitudes toward the lung screen were assessed with: (1) “Do you believe undergoing this lung screening is important for my health and vitality?” (from “not at all important” to “extremely important”); and (2) “How stressful is undergoing this lung screening for you? (from “not at all stressful” to “extremely stressful”).

### Statistical Analysis

All analyses were conducted using SAS Software 9.4 (SAS Inc., Cary, NC, United States). Responses were examined as counts (percent) and modeled using generalized linear modeling and mixed modeling, when appropriate with the FREQ and GLIMMIX procedures.

## Results

### Smoking Behavior

Participants smoked a median of 40 years with a median of 16 cigarettes per day. Shown in Table 1, cigarette smoking was the most prevalent (86.6% smoked every day). Interestingly, 98.2% (373/380) of every-day cigar smokers were also every-day cigarette smokers.

**TABLE 1:** Smoking behavior and smoking cessation attitudes.

Outcome	% (Frequency)/Median (IQR)
*Smoking behavior*	
Number of years smoked	*M* = 40 (35–46)
Number of cigarettes smoked per day smoking	*M* = 16.5 (10–20)
Cigarette smoking frequency	
Every day	86.6% (1,175/1,357)
Some days	7.3% (99/1,357)
Not at all	6.2% (83/1,357)
Cigar smoking frequency	
Every day	30.1% (384/1,276)
Some days	5.2% (63/1,276)
Not at all	64.8% (827/1,276)
e-Cig smoking frequency	
Every day	2.6% (33/1,294)
Some days	5.1% (66/1,294)
Not at all	92.3% (1195/1,294)
*Interesting in quitting*	
Not at all interested	8.6% (110/1285)
Somewhat interested	19.6% (252/1,285)
Moderately interested	13.4% (172/1,285
Interested	21.3% (274/1,285)
Very interested	37.1% (477/1,285)
*Quitting thoughts*	
Not thinking about quitting	25.9% (337/1,299)
Thinking about quitting in next 6 months	37.7% (490/1,299)
Thinking about quitting in next 30 days	36.3% (472/1,299)
*Confidence in quitting in 1 year*	
Not very confident	28% (363/1,279)
Somewhat confident	38% (479/1,279)
Confident	16% (201/1,279)
Very confident	19% (236/1,279)
*Inherent ability to quit*	
Not at all (I am totally unable to change my smoking habits)	8.4% (113/1,353)
A little	22% (295/1,353)
Somewhat	37% (500/1,353)
Mostly	19% (253/1,353)
Completely (I am totally able to change my smoking habits)	14% (192/1,353)

### Attitudes About Quitting and Planning to Quit

**FIGURE 1:**
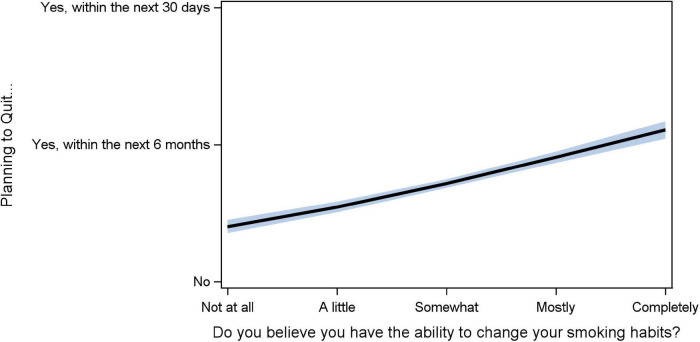
Association between perception of inherent (implicit) ability to quit and planning to quit. The *Y*-axis is planning to quit (Not, within 6 months, with 30 days). The *X*-axis is the level of belief in the patient’s implicit ability to quit (0–4: not at all, a little, somewhat, mostly, completely). The black line is the fitted slope for responses. The blue shadow is the 95% confidence band.

Only 8.4% (113/1,353) of current smokers believe they do not have inherent ability to stop smoking, while 22% (295), 37% (500), 19% (253), and 14% (192) believe they have a little, somewhat, mostly, or completely inherent ability to quit. For every one-unit increase in ability to quit, the odds of planning to quit increased 49% (OR: 1.492, 95% CI [1.4, 1.6], *p* < 0.0001; Figure 1).

**FIGURE 2:**
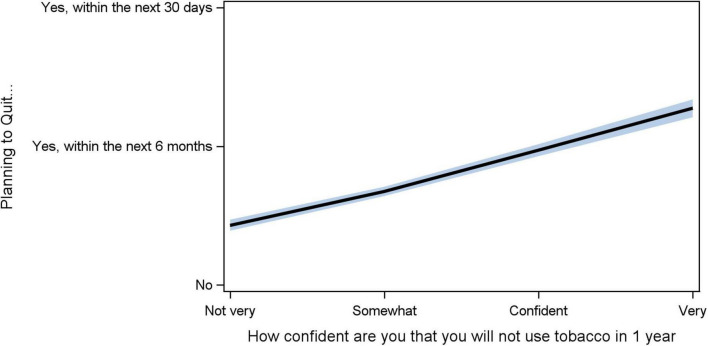
Association between confidence in not using tobacco (self-efficacy) and planning to quit. The *Y*-axis is planning to quit (Not, within 6 months, with 30 days). The *X*-axis is the level of confidence (0–3: not very, somewhat, confident, very). The black line is the fitted slope for responses. The blue shadow is the 95% confidence band.

Although 28% (363/1,279) of current smokers are not very confident they can quit, 38% (479), 16% (201), and 19% (236) are somewhat confident, confident, and very confident they can quit, respectively (Table 1). For every one-unit increase in confidence, the odds of thinking about quitting increased 86% (OR: 1.859, 95% CI [1.7, 2.0], *p* < 0.0001; Figure 2).

Finally, on the contemplation change ladder, only 6.5% (77/1,192) of patients report no thought of quitting while 3.6% (43) reported considering quitting someday and 18% (219) reported believing they should quit but are not ready to quit. Interestingly, 20% (243) report starting to think about changing their smoking patterns and 23% (276) report having reduced the amount they smoke and/or seeking enrolling in a program. For every one-unit increase in contemplation, the odds of thinking about quitting increased 36% (OR: 1.355, 95% CI [1.3, 1.4], *p* < 0.0001; Supplementary Figure 1).

### Outcome of the Report and Smoking Reduction

**FIGURE 3:**
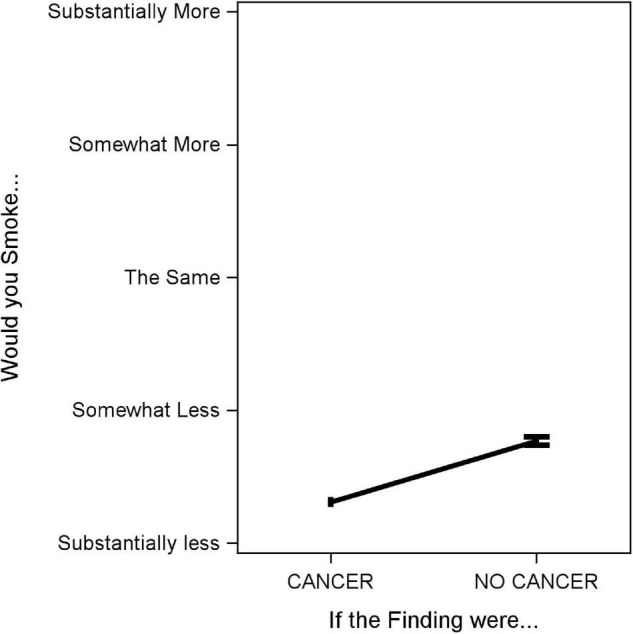
Association between lung screen result showing cancer/not cancer and anticipated smoking behavior. The *Y*-axis is planned smoking behavior (0–4: smoking substantially less, somewhat less, the same, somewhat more, substantially more). The *X*-axis is the hypothetical finding result (cancer versus no cancer). The black line is the delta between the mean estimate for each finding with 95% confidence intervals.

Patients reported they would smoke less if told their screen was positive for cancer compared to negative (0.31, 95% CI [0.27, 0.34] vs. 0.77, 95% CI [0.72, 0.81]), where 0 is “smoke substantially less” and 4 is “substantially more”, *p* < 0.0001; however, on average, patients reported the desire to smoke less regardless of the screen showing cancer or not (Figure 3).

### Interest in Quitting and Quitting Thoughts

When assessing interest in quitting, 8.6% reported being not at all interested in quitting, while 19.6%, 13.4%, 21.3%, and 37.1% were somewhat interested, moderately interested, interested, or very interested in quitting, respectively–91.4% were interested to some degree in quitting. Similarly, 25.9% were not seriously thinking about quitting, while 37.7% were thinking about quitting in the next 6 months and 36.3% were thinking about quitting in the next 30 days–74.1% were seriously thinking about quitting in the next 6 months or sooner (Table 1). The relationship between interest in quitting and planning to quit is illustrated in Supplementary Figure 2. For every one-unit increase in interest in quitting, the odds of thinking about quitting increase 96% (OR: 1.959, 95% CI [1.840, 2.084], *p* < 0.0001).

### Attitudes Toward Lung Screen Itself

When asked how important the lung screen was to their health and vitality, 69% (887/1,283) reported it was extremely important, 15% (195) moderately important, 10% (134) somewhat important, 3% (44) a little important and 1.8% (23) said not at all important. When asked how stressful it was to get a lung screen, 48% (635/1,326) said not stressful at all while 27% (352), 13% (175), 6% (83), and 6% (81) said it was a little, somewhat, moderately, and extremely stressful, respectively.

## Discussion

As the number of smokers who undergo lung screening increases due to relaxed guidelines (3), more consideration should be given to the smoking behavior and smoking cessation attitudes in this population. We observed that current smokers who present for lung cancer are heavy smokers with a median of 32 pack-years. The vast majority of current smokers smoke cigarettes every day, and a sizeable minority use cigars conterminously daily; very few report smoking e-cigarettes.

Nearly all current smokers were at least somewhat interested in quitting smoking, with a modal response of “very interested” (37.1%). Furthermore, most patients say that they will smoke less regardless of whether the lung screen shows they have cancer or not, which suggests interest in cessation is not just anticipatory of the possibility of having cancer. Put differently, patients report planning to make behavioral changes in the coming days regardless of their lung cancer report. As discussed below, this finding has important implications for smoking cessation among a very high-risk group of smokers. These findings stand in contrast to some qualitative studies where there is less evidence supporting patient’s interest in quitting (5, 9). One reason for the discrepancy might be due to sample differences. In the present study, we included nearly all patients who presented for lung cancer screening in the state of Rhode Island over the course of a year. By contrast, Golden et al. (5) included patients *offered*, but not necessarily enrolled in, lung cancer screening while Zeliadt et al. (9) sample was restricted to patients at the Veterans Health Administration.

### Smoking Cessation Interventions

Heavy smokers undergoing a lung cancer screen should receive evidence-based smoking cessation feedback. This is important not just because of the health benefits of quitting smoking, but because the population reports being receptive to and interested in reducing their tobacco use. Additionally, while many heavy smokers have presumably received cessation advice in the past, the lung cancer screening context is a unique opportunity because such advice will likely be seen as less divorced from lung cancer or other smoking-related consequences. Thus, consistent with suggestions by others (6, 7, 12), lung cancer screening may represent a propitious time to engage patients in smoking cessation given both the timing, and as shown in our data, their interest in cessation.

### A One-Size Fits All Approach to Cessation?

Future trials should consider tailoring cessation interventions to patients’ attitudes. Most patients want to quit, but there is variability in whether patients believe they have the inherent ability to stop smoking, and their confidence in smoking cessation. Different types of interventions might be needed to address this full spectrum of attitudes. For instance, patients with high perceived control and confidence may simply need to be connected with resources (e.g., tobacco replacement therapy), while those with low perceived control and confidence may need cognitively-based therapy sessions to change attitudes before behavioral treatment will be effective. Tailoring in this fashion, which has also been recommended elsewhere (16), is consistent with the goals of precision medicine. While much prior work on precision medicine for smoking cessation has focused on biological factors (13, 14), psychological constructs are more feasible to measure and therefore may have more promise for wide-spread use. Additionally, since some treatments (e.g., counseling) are more resource intensive than others, tailored interventions could be created in a manner that maximizes cost-efficiency.

### Limitations

Limitations of the present study include the cross-sectional design and reliance on self-report data. Also, due to the nature of setting, the assessment was brief so constructs of interest were not examined using lengthier, and perhaps more validated, measures.

### Conclusion and Implications

Smokers who receive a lung cancer screen are interested in smoking cessation as a group, and report planning to reduce how much they smoke. Lung cancer screening visits, follow-ups, and result letters represent an enormous opportunity to connect these patients with smoking cessation resources and deliver tailored cessation interventions.

## Data Availability Statement

The data analyzed in this study is subject to the following licenses/restrictions: The dataset is not publicly available. Requests to access these datasets should be directed to GB, gbaird@lifespan.org.

## Author Contributions

MB, GB, and SA conceived of the study. MB and GB drafted the manuscript. MB, GB, and TH drafted the survey. MB, GB, KO, AA, and SH extracted the data. All authors reviewed, contributed to the manuscript, and approved the submitted version.

## Conflict of Interest

The authors declare that the research was conducted in the absence of any commercial or financial relationships that could be construed as a potential conflict of interest.

## Publisher’s Note

All claims expressed in this article are solely those of the authors and do not necessarily represent those of their affiliated organizations, or those of the publisher, the editors and the reviewers. Any product that may be evaluated in this article, or claim that may be made by its manufacturer, is not guaranteed or endorsed by the publisher.
