# Supplementary material for: Heavy Smoking Patients Receiving a Lung Cancer Screen Want to Quit: A Call for Tailored Cessation Interventions
Source: Front Med (Lausanne). 2022 May 11;9:816694. doi: 10.3389/fmed.2022.816694 (PMC9130603; doi:10.3389/fmed.2022.816694)
Supplement: Supplementary file 1 [file Data_Sheet_1.docx]

**Heavy smoking patients receiving a lung cancer screen want to quit: A call for tailored cessation interventions**

Bernstein et al., 2022. *Frontiers in Medicine*

SUPPLEMENTAL MATERIALS


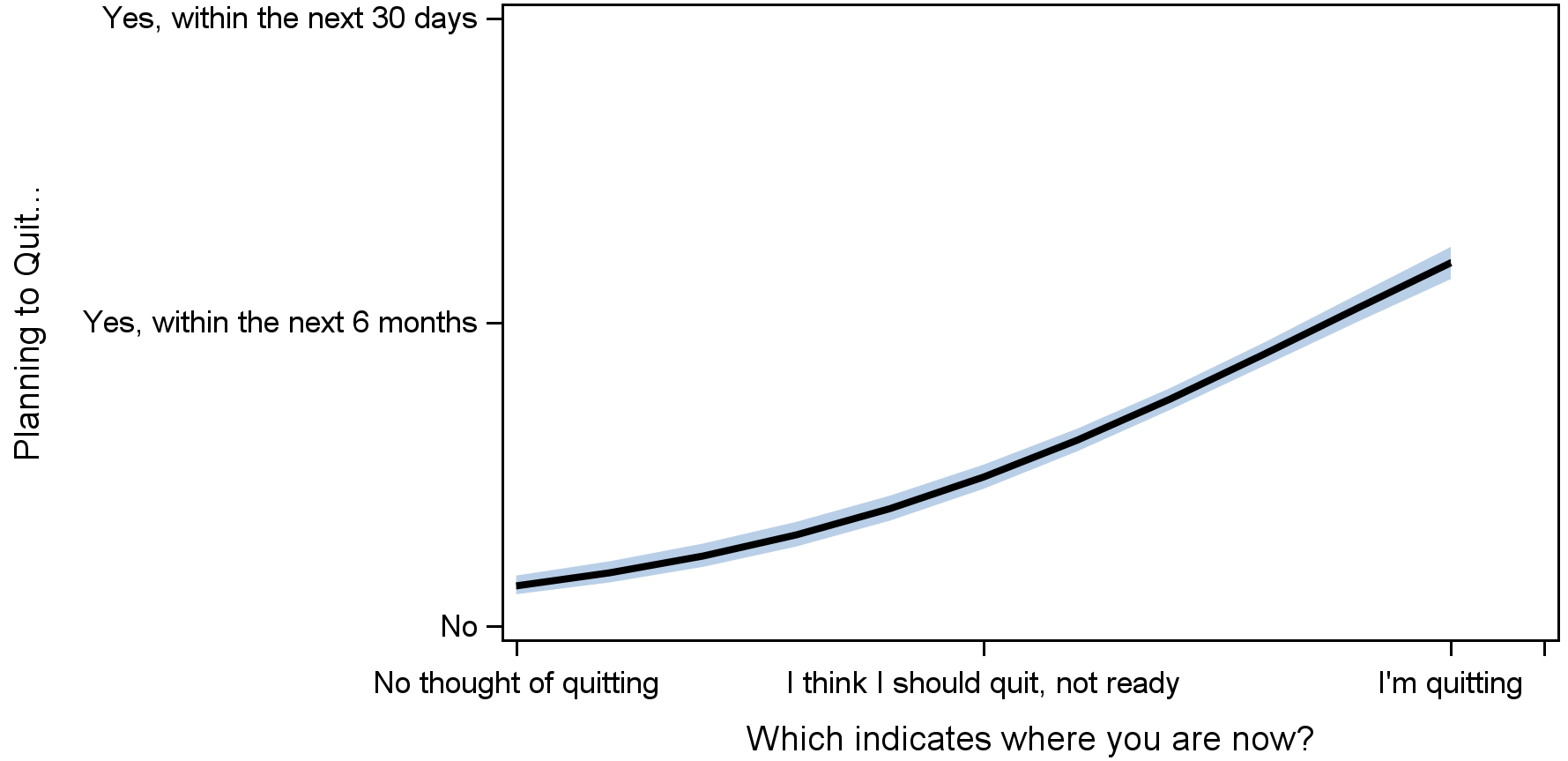


**Figure S1.** **Association between thought of quitting and planning to quit.** The Y-axis is planning to quit (Not, within 6 months, with 30 days). The X-axis is thought of quitting (0-10: No thought of quitting (0), thought of quitting but no plan (2), taking steps to quit (10). The black line is the fitted slope for responses. The blue shadow is the 95% confidence band.


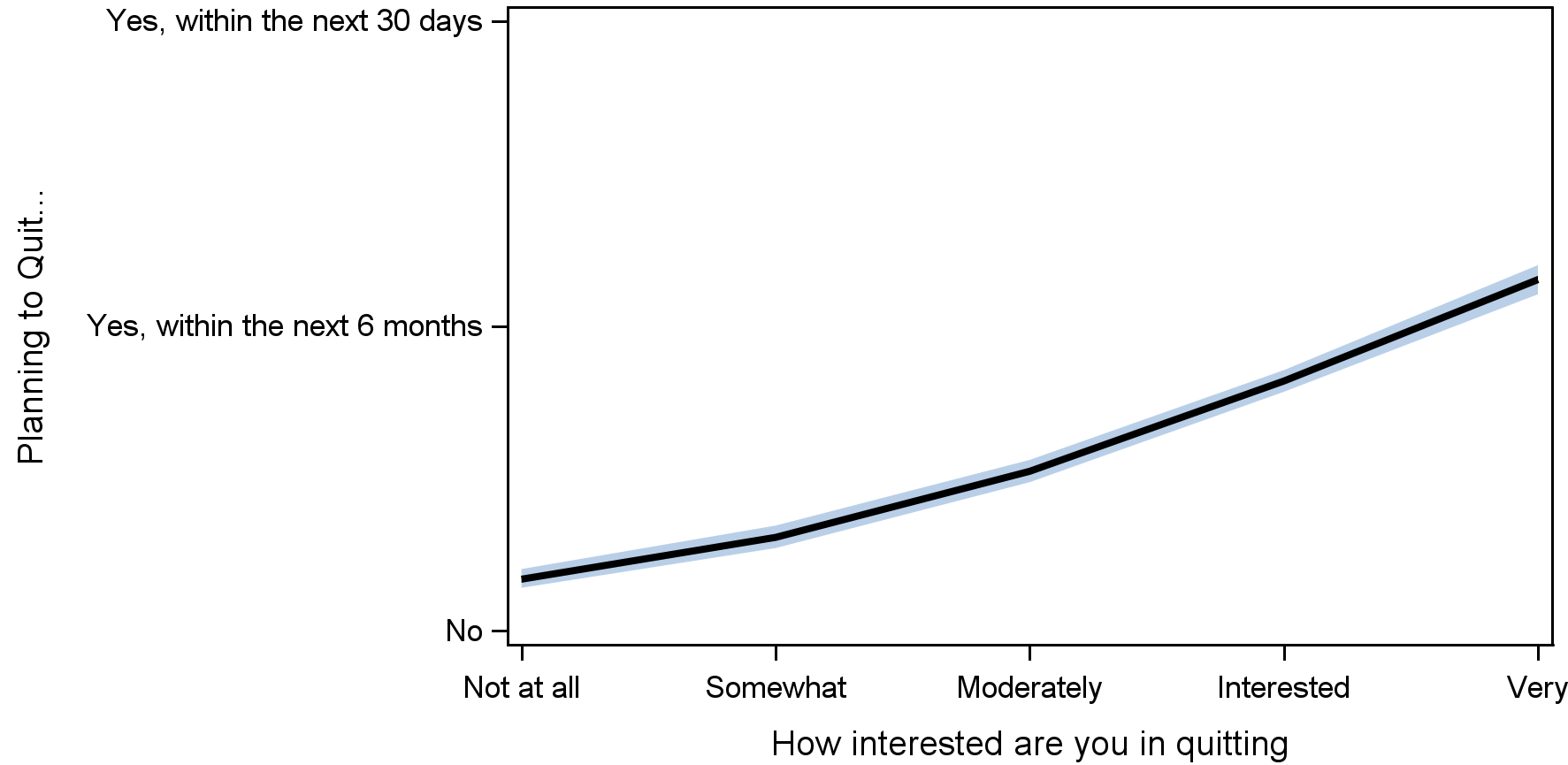


**Figure S2.** **Association between interest in quitting and planning to quit.** The Y-axis is planning to quit (Not, within 6 months, with 30 days). The X-axis is the level of interest in quitting (0-4: not at all, somewhat, moderately, interested, very). The black line is the fitted slope for responses. The blue shadow is the 95% confidence band.
